# Action Observation Therapy for Upper Limb Recovery in Patients with Stroke: A Randomized Controlled Pilot Study

**DOI:** 10.3390/brainsci11030290

**Published:** 2021-02-26

**Authors:** Mauro Mancuso, Serena Di Tondo, Enza Costantini, Alessio Damora, Patrizio Sale, Laura Abbruzzese

**Affiliations:** 1 Physical and Rehabilitative Medicine Unit, NHS-USL Tuscany South-Est, Via Senese 169, 58100 Grosseto, GR, Italy; enzacostantini@alice.it; 2 Tuscany Rehabilitation Clinic, Montevarchi, Piazza del Volontariato 2, Montevarchi, 52025 Arezzo, AR, Italy; serenaditondo@gmail.com (S.D.T.); alessio.damora@gmail.com (A.D.); laura.abbruzzese@libero.it (L.A.); 3 Sant’Isidoro Hospital, FERB Onlus, Via Ospedale 34, 24069 Trescore Balneario, BG, Italy; patrizio.sale@gmail.com

**Keywords:** stroke, action observation, rehabilitation, mirror neurons, upper limb

## Abstract

Due to the complexity of the interventions for upper limb recovery, at the moment there is a lack of evidence regarding innovative and effective rehabilitative interventions. Action Observation Training (AOT) constitutes a promising rehabilitative method to improve upper limb motor recovery in stroke patients. The aim of the present study was to evaluate the potential efficacy of AOT, both in upper limb recovery and in functional outcomes when compared to patients treated with task oriented training (TOT). Both treatments were added to traditional rehabilitative treatment. Thirty-two acute stroke patients at 15.6 days (±8.3) from onset, with moderate to severe upper limb impairment at baseline following their first-ever stroke, were enrolled and randomized into two groups: 16 in the experimental group (EG) and 16 in the control group (CG). The EG underwent 30 min sessions of AOT, and the CG underwent 30 min sessions of TOT. All participants received 20 sessions of treatment for four consecutive weeks (five days/week). The Fugl-Meyer Assessment for Upper Extremity (FMA-UE), Box and Block Test (BBT), Functional Independence Measure (FIM) and Modified Ashworth Scale (MAS) were administered at baseline (T_0_) and at the end of treatment (T_1_). No statistical differences were found at T_0_ for inclusion criteria between the CG and EG, whereas both groups improved significantly at T_1_. After the treatment period, the rehabilitative gain was greater in the EG compared to the CG for FMA-UE and FIM (all *p* < 0.05). Our results suggest that AOT can contribute to increased motor recovery in subacute stroke patients with moderate to severe upper limb impairment in the early phase after stroke. The improvements presented in this article, together with the lack of adverse events, confirm that the use of AOT should be broadened out to larger pools of subacute stroke patients.

## 1. Introduction

It is well known that stroke is the leading cause of death [1] and one of the greatest causes of long-term motor disability in adults [2]. More than 80% of patients show upper limb impairment after a stroke, and approximately half of them maintain a reduction in voluntary movements of the upper limbs. Verticalization and ambulation are the first goals that people ask to reach after a stroke [3], but the improvement of these abilities is not enough to restore social participation in patients when they return home. In fact, even if gait and mobility are recovered after rehabilitation, they are not enough to give back autonomy in daily activities, which are based on dexterity of the upper limbs. Many daily activities, including eating, dressing, and washing, are based on the ability to move and coordinate the arms, hands, and fingers. Limb impairment affects the abilities of patients to carry out daily activities, resulting in huge financial burdens for patients, families, and society [4]. 

Due to the role that upper limb impairment plays in determining limitation of daily activities [5], many rehabilitative strategies have been developed to promote the recovery of the upper limbs. However, due to the complexity of the intervention in recovery of the upper limbs, there is presently a lack of evidence about more effective rehabilitative interventions [6]. Moreover, patients also show concomitant impairment of cognitive functions, which impedes motor recovery [7]. Impairments such as language [8] and spatial cognition [9] hamper the recovery of patients, leading to a poor outcome [7,10]. It seems likely that the ability to restore motor functions is mainly based on cortical plasticity, strictly connected to learning capacities [11]. 

In recent years, many studies have shown that the reorganization of the damaged cortical areas can be improved by observation of actions from daily activities [12]. The involvement of the Mirror Neuron System (MNS) in motor learning has allowed the development of a new rehabilitation approach, called Action Observation Training (AOT), in which patients are asked to carefully watch a video clip on a screen and then to imitate the actions seen [13,14,15,16].

A recent meta-analysis has shown that AOT appears to be effective in improving upper limb motor recovery [17], whereas other articles [18,19,20,21] have highlighted how efficacy seems to be related to different application modalities. In fact, passive and active upper limb movements seem to increase motor recovery, due to the effects on somatosensory input, motor planning, soft tissue properties, and spasticity [18]. The effectiveness improves when actions are divided into three to four distinct motor acts. For example, the AOT of drinking water should be separated into three consecutive motor acts: pouring water into the glass, reaching for the glass and bringing it to the mouth. In addition, perspective plays a relevant role, as frontal viewing seems to be the most effective compared to other points of viewing [18]. Patients tend to perform better when they repeat the same movements using the same tools observed in the video, and the effect is stronger when they hear the sounds of the observed actions [19]. Congruence of observed action with physical training enhances the effects of motor training [20]. The timing between screen viewing and repetition is also significant [21].

Among the factors that influence the efficacy of Action Observation Treatment, a fundamental role is played by the difficulty in transferring the functional gains of the treatment to everyday life. A recent systematic review and meta-analysis addressed the importance of a more comprehensive view of functional gains, examining the effects of priming on task-oriented training on upper extremity outcomes [22]. The authors highlighted the need to identify the role of Action Observation priming and Task-Oriented Training (TOT), which involves functional activities executed with the upper limbs, since there is insufficient evidence to clarify the effects of the association of this type of priming with TOT.

In this regard, the literature data show the importance of a more comprehensive view of functional gains that allows clinicians to determine the optimal treatment for stroke patients [23,24].

Thus, the aim of the present study was to evaluate the potential efficacy of AOT both on upper limb recovery and global functional outcome, as compared to TOT, based on Activities of Daily Livings (ADLs) in a sample of stroke patients that was in the early phase after stroke. We expected higher motor recovery and functionality of the paretic upper limbs and better independence in daily life activities in the group of patients that underwent AOT.

## 2. Materials and Methods

### 2.1. Participants

Due to a lack of information upon which to base the sample size, we adopted the recommendation for a pilot study of a minimum of 12 subjects per group [25]. In this pilot study, we recruited a sample of 32 stroke patients admitted to the neurological rehabilitation unit of the Tuscany Rehabilitation Clinic.

Patients enrolled in the sample were of both genders, of any education level, and aged from 18 to 90 years old, having had their first-ever stroke, with a unilateral cerebral lesion, upper limb impairment, and at a maximum of 30 days from onset. The ability to understand spoken language (Token Test score higher than 8) [26,27] was required for all the patients.

All enrolled patients with unilateral brain lesions following their first-ever stroke were clinically evaluated by a neurologist and underwent a CT brain scan.

Patients with subarachnoid haemorrhage, severe neglect at the Star Cancellation from the Behavioural Inattention Test (a score lower than 51) [28], impaired comprehension (a score lower than 8 in the Token Test) [26,27], ideomotor apraxia^,^ [29], cognitive decline (a score lower than 23.8 in the Mini Mental State Examination) [30], or a history of endogenous depression or severe psychiatric disorders for which patients needed chronic pharmacotherapy were excluded from the study. In addition, patients with severe visual deficits, diagnosed by means of the eye field examination of the National Institutes of Health Stroke Scale (NIHSS) [31] subtest, were excluded.

The Local Ethical Committee approved the study protocol (protocol code 17117; date of approval 20/04/2020). The study was registered to ClinicalTrials.gov, with the number NCT04604171. All the participants were asked to carefully read and sign the informed consent.

All procedures conformed to the World Medical Association declaration of Helsinki (2016).

### 2.2. Procedure

We conducted a blind, randomized, controlled pilot study. Participants were randomly allocated to the experimental group (EG) or to the control group (CG). A computer-generated random number was used to allocate participants arbitrarily to the action observation training group or the control group. The randomization procedure and assignment were managed by an independent researcher who was not involved in evaluation of the participants. Sixteen patients were assigned to EG and sixteen to CG.

Each participant underwent clinical assessments at T_0_ time (the same day the AO treatment began) and at T_1_ time (after four weeks).

All assessments were performed by trained researchers not involved in the treatment administration and blinded to the patient’s allocation.

All subjects underwent rehabilitative treatment for 4 consecutive weeks, 5 days per week. Each session consisted of 60 min of conventional treatment (CT) per day as well as 30 min of AOT for the EG or 30 min of TOT for the CG. The Fugl-Meyer Assessment for Upper Extremity (FMA-UE) was used as the primary outcome measure. Secondary outcomes included the Box and Block Test (BBT), the Modified Ashworth Scale (MAS), and the Functional Independence Measure (FIM).

#### 2.2.1. Clinical Scales

- Canadian Neurological Scale (CNS) [32]: this provides a standardized neurological assessment of cognitive and motor function in stroke patients, giving a quantification of severity. It includes the following assessments:
  1. Level of consciousness;
  2. Orientation;
  3. Aphasia;
  4. Motor strength.
  Assessment of motor functions is administered with different instructions, depending on the level of comprehension impairment [33]. The total score of CNS ranges from 1.5 to 11.5. Lower scores correspond to higher severity.
- Statistical analysis weres wase a ted The Bamford Classification [34] allows clustering of patients with cerebral infarction according to some distinctive features, placing them into the following four groups on the basis of the signs and symptoms: Lacunar Infarcts (LACI), Total Anterior Circulation Infarcts (TACI), Partial Anterior Circulation Infarcts (PACI), and Posterior Circulation Infarcts (POCI).

#### 2.2.2. Primary Outcome Measure

- Fugl-Meyer Assessment for Upper Extremity (FMA-UE) [35]: the scale, which has good psychometric properties [36], is a quantitative measure of motor impairment in post-stroke hemiplegic patients. Items are scored on a 3-point ordinal scale (0 = cannot perform; 1 = partially performs; 2 = fully performs). The upper limb section (FMA-UE) includes shoulder, elbow, and wrist flexion and extension cooperative movement, wrist joint stability, coordination ability, and speed of small joint movement. The four domains assessed include motor function, sensation, passive joint motion, and joint pain. For this study we only used the motor function subscale. The total score of FMA-UE motor function ranges from 0 to 66 [37]. The MCID score for upper extremity motor recovery among patients with subacute stroke is 9 to 10 on the FMA-UE [38].

#### 2.2.3. Secondary Outcome Measures

- Box and Block Test (BBT) [39]: the BBT assesses unilateral gross manual dexterity in stroke subjects. Patients sit at a table, facing a rectangular box that is divided into two sections of equal dimensions. One of the two compartments contains 150 colored wooden cubes, measuring 2.5 cm in width. The subject is asked to move as many blocks as possible, one at a time, from one section to the other for a period of 60 s. The final score is computed by counting the number of blocks moved during the one-minute trial period. Healthy adults aged 20 and up have been found to move around 75 cubes ± 9.1 within one minute, without any significant difference between the dominant and non-dominant hand [40]. Its reliability and validity are satisfactory for stroke patients [41]. The MCID score for BBT corresponds to 5.5 blocks per minute [42].
- Functional Independence Measure (FIM) [43,44]: the purpose of this scale is to assess the patients physical, psychological, and social functions. It includes self-care, eating, grooming, bathing, dressing, toileting, swallowing, sphincter control, mobility, transfer, and locomotion. The scale is composed of 18 items: 13 items are in physical domains and 5 items are related to cognition. Motor items measure self-care, sphincter control, locomotion, and transfers. Cognitive items evaluate the subject’s communication abilities and social cognition. Based on the level of independence, each item is scored from 1 to 7. The lower score indicates total dependence and the higher represents complete independence. The total score ranges from 18 to 126. The total FIM score indicates the level of disability and the burden of their caregiver. The FIM has good reliability, validity, and responsiveness [45]. The MCID score for FIM total score is 22 [46].
- Modified Ashworth Scale (MAS) [47]: this is a six-point ordinal scale for grading the resistance encountered during passive muscle stretching [48]. The scale assesses spasticity as follows: 0 = normal muscle tone; 1 = slight increase in muscle tone at the end of the range of motion (ROM) when limb is moved; 1+ = slight increase in muscle tone, manifested by a catch, followed by minimal resistance throughout the remainder (less than half) of the ROM; 2 = more marked increase in muscle tone, but limb easily flexed; 3 = considerable increase in muscle tone; and 4 = limb rigid in flexion or extension. In our study, we tested: shoulder adductors and abductors, elbow flex-extensors, forearm prone-supinators, wrist flex-extension, finger flex-extension, and thumb flex-extension. A decrease of one point on the MAS scale reflects a clinically significant improvement [49].

#### 2.2.4. Treatments Adopted in the Study

- AOT: Action Observation Training. AOT [50] is composed of 20 different videos of daily activities (actions) carried out with the upper limbs. Patients undergo only one task per day for 20 sessions, starting from the easiest. Each action (unimanual or bimanual) is observed from a first-person perspective. Actors in the videos are young non-disabled people, either men or women. Patients are asked to carefully observe the videos in order to prepare themselves to imitate the presented actions, while the therapist consistently holds the patient’s attention with verbal feedback. At the end of each sequence, the therapist prompts the patient to perform the same movement with the paretic upper limb over a time period of 2 min, providing verbal instructions or help, if needed. Each session lasts about 30 min (3 min of sequence observation and 2 min of action performance for 3 motor sequences, repeated twice).
- TOT: Task Oriented Training. In TOT patients perform functional activities with the upper limbs, using the same objects as AOT, in both unimanual and bimanual modalities, without watching the video beforehand. The therapist only provides verbal instructions and feedback, avoiding demonstrative or imitative indications. The therapist can passively support the movement if patients are completely unable to perform the actions. When necessary, the therapists can also actively facilitate the upper limb movement if patients are unable to correctly perform the actions. Based on the patient’s level of motor ability and progress, the levels of movement and task difficulty can be adjusted accordingly. Patients undergo one task per day for 20 sessions. Each session lasts about 30 min.
- CT: Conventional Treatment. Conventional treatment consists of a range of different patient-tailored interventions, not involving AOT or TOT objects, selected by the physiotherapist on the basis of the functional level of the patient. Treatment sessions include training for transfers, mobility, walking up and down steps, balance tasks, and tailored functional tasks for the upper limbs (unimanual and bimanual). Moreover, it is also provided for joint and soft tissue mobilization and specific sensory stimulation and can include exercises to increase strength, both for lower and upper limbs. Each session lasts about 60 min, 20 min of which are spent on treating the upper limb.

### 2.3. Statistical Analysis

Due to small sample size, non-parametric tests were used.

We used Mann-Whitney U test and Chi-square analyses to assess the homogeneity of the sample before the study, according to demographic and clinical data, as appropriate.

To determine statistical significance of within-group improvement from admission to discharge, pre-treatment and post-treatment scores were analyzed using the Wilcoxon signed-rank test.

Comparisons between groups were carried out using the rehabilitative gain, which was calculated as the difference between the post-treatment score and the baseline, divided by the difference between the maximum scoring of the test and the score obtained by the patient at the baseline. This index indicates the percentage of the improvement compared to the maximum obtainable improvement. Between-group comparisons were carried out by means of the Mann-Whitney U test.

Furthermore, the minimum clinically important difference (MCID) was used for the variables in which the groups differed in terms of rehabilitative gain. The MCID is defined as “the smallest difference in the score in the domain of interest which patients perceive as beneficial and which would warrant, in absence of troublesome side effects and excessive cost, a change in the patient’s management” [51]. The Chi-square test was used to compare the number of patients in the EG and in the CG that overcame the MCID value for the variables in which groups differed in terms of rehabilitative gain.

The alpha level for significance was set at *p* < 0.05 for the first level of analysis. Statistical analysis was carried out using the Statistical Package for the Social Sciences (SPSS) software, version 20.0 (SPSS Inc., Chicago, IL, US).

## 3. Results

We assessed and screened a total of 85 voluntary patients, but just 32 of them met the inclusion criteria. All patients were, on average, at 15.6 days (± 8.3) from onset.

The 32 patients enrolled in this pilot study were randomly assigned to the EG (16 subjects) and CG (16 subjects). All patients were right-handed. No dropouts were recorded during the treatment, and all subjects fulfilled the protocol (compliant subjects: n = 32). FMA-UE motor function was selected as the primary outcome measure.

Demographic and baseline clinical characteristics are shown in Table 1.

No significant differences at baseline were observed between the groups with regard to age, gender, localization of brain lesions (Bamford classification), global cognitive level (MMSE), and stroke severity (CNS) (all *p* > 0.05). The control group had a higher prevalence of right hemispheric lesions (*p* < 0.05). No significant differences between the EG and the CG were detected at baseline in FMA motor function (Z = −0.24; *p* > 0.05), BBT (*Z* = −0.43; *p* > 0.05), and FIM (*Z* = −0.67; *p* > 0.05). In MAS, the CG showed a higher score at T_0_ compared to the EG (*Z* = −2.8; *p* < 0.05).

Regarding the primary outcome measure, on average both groups showed a significant improvement from T_0_ to T_1_ in FMA motor function (*Z* = −4.5; *p* < 0.001). Within-group comparisons between baseline and post-treatment scores showed a significant statistical difference in the EG and CG from T_0_ to T_1_ in FMA motor function (EG: *Z* = −3.5; *p* < 0.001; CG: *Z* = −2.8; *p* < 0.05). Furthermore, the EG showed significantly higher scores in FMA motor function in terms of rehabilitative gain with respect to the CG (*Z* = −2.1, *p* < 0.05).

In secondary outcome measures, on average both groups showed a significant improvement from T_0_ to T_1_ in BBT of the paretic arm (*Z* = −3.8; *p* < 0.001) and in FIM total score (*Z* = −4.7; *p* < 0.001). Both the EG and the CG showed a significant statistical difference from T_0_ to T_1_ in within-group comparisons between baseline and post-treatment scores in BBT of the paretic arm (EG: Z = −2.93; *p* < 0.05; CG: *Z* = −2.5; *p* < 0.05) and in FIM (EG: *Z* = −3.5; *p* < 0.001; CG: *Z* = −3.2; *p* < 0.001). Regarding rehabilitative gain, comparisons between add-on task-oriented training and add-on AOT showed significantly higher FIM scores in the EG compared to the CG (*Z* = −2.9; *p* < 0.05).

No evidence of a change in the spasticity of the shoulder, elbow, and wrist were detected in either the treatment group or the control group (all *p* > 0.05).

Table 2 shows median, interquartile range (IQR), and significance of within-group and between-group comparisons and of the rehabilitative gain.

The Chi-square test, which was used to compare the number of patients in the EG and the CG that overcame the MCID value for the FMA motor function and FIM variables, did not show significant difference between groups (all *p* > 0.05). Table 3 reports the difference in scores between baseline and post-treatment assessments in the experimental group and in the control group for the Fugl-Meyer Assessment for Upper Extremity (FMA-UE) and the FIM scales, showing the patients that overcame the MCID for such scales.

## 4. Discussion

The aim of the present pilot study was to evaluate the effects of AOT compared to TOT, both on upper limb recovery and on functional outcome, in a sample of patients after a stroke.

Starting from the evidence that AOT promotes motor learning, improving outcomes after rehabilitation [17,52], we studied a sample of post-stroke patients with impairment of the upper limb, comparing AOT to a very similar treatment in TOT. The motor and functional outcomes of 32 acute stroke patients were assessed in order to detect differences in the recovery of the upper limbs.

All patients had a significant improvement in all of the administered scales, but the EG had a significantly higher rehabilitative gain in upper limb functioning compared to the CG. Previous studies [50,53,54,55] have shown that action observation training, combined with task-oriented training, produces greater improvements in motor performance of the action observation group with respect to the control group, consistent with findings in the present study. On the basis of these results, it has been suggested that action observation training, combined with physical practice, may be a useful method by which to recover limitations in motor function and improve activity levels in patients in the subacute post-stroke stage.

In our study AOT was compared to training in which patients performed functional activities with the upper limbs, using the same objects as for AOT but without watching the video beforehand. Our results might suggest that the presence of the video, the repetitiveness, and the systematic nature of AOT may induce neural plasticity by promoting activation of the damaged motor circuits and by providing access to multiple brain areas, thus facilitating motor relearning. Furthermore, AOT proposes even more complex motor sequences with respect to the usual use of the objects. In fact, AOT movement sequences are standard, regardless of the patient’s level of functionality, whereas in task-oriented training the level of difficulty is decided by the therapist in relation to the functional level of the patients.

A point of interest of our study is that the improvement observed in the action observation group reached a higher value than the minimum clinically important difference (MCID) for motor function and functional outcome scores. Thus, the difference from admission to discharge in motor function and functional outcome scores was perceived as beneficial enough by the patients to warrant a change in the patient’s management.

With regard to motor function, FMA was used as the primary outcome measure, since it is one of the most comprehensive quantitative measures of motor impairment following stroke and has been recommended for clinical trials related to stroke rehabilitation [56]. Furthermore, the FMA is a valid and reliable instrument to use in clinical settings [57]. The MCID score for upper extremity motor recovery among patients with subacute stroke is 9 to 10 on the FMA-UE [38] and 22 on the FIM total score [46]. In our sample, the significant difference in the rehabilitative gain of the experimental group compared to the control group corresponds to a clinically important difference in the recovery of motor function and independence in daily life activities. In fact, in the group of patients trained with AOT, a mean difference score of 14.1 between baseline and post-treatment assessments in FMA-UE scores was detected, underlying the fact that 12 patients in the experimental group perceived a beneficial difference in the score in the motor function domain compared to four patients in the control group (mean difference score CG = 6.1). In addition, in the FIM total score the difference between baseline and post-treatment assessments overcame the MCID score of 22 in 13 patients that underwent AOT compared to seven that underwent task-oriented training (mean difference score EG = 36.4 versus CG = 19.9). This is in accordance with one previous study [58], in which the authors found a higher number of patients achieving the MCID values on the motor and functional outcomes in the action observation therapy group. Thus, the addition of AOT is associated with clinically meaningful improvements and may be helpful in improving upper extremity functions and daily life autonomy in stroke patients.

Moreover, the efficacy of AOT in recovering the upper limbs in patients after a stroke is supported by recent neurophysiological evidence that has shown how the observation and the execution of movements performed by healthy people improve the functional recovery of the upper limbs and promote autonomy in daily life activities [59,60].

The neurophysiological correlation of this effect was found in the Motor Evoked Potentials (MEPs), a method that allows clinicians to judge the change of central nervous system plasticity induced by rehabilitation therapy in stroke patients. An effect of AOT on motor nerve excitability was found as demonstrated by an improvement of the Motor Evoked Potentials (MEPs) latency and of the functionality of the upper motor neuron in the experimental group [59]. Furthermore, a recent review demonstrated that action observation training has a moderate effect on arm and hand motor outcomes, a moderate to large effect on walking outcomes, a large effect on gait velocity, and a moderate to large effect on daily functions [61]. With regard to gait function recovery, it has been observed that after four weeks of AOT, patients have significant improvement in step length, stride length, cadence, and velocity [62].

A recent study demonstrated a greater improvement in the Functional Independence Measure in the group of patients that underwent AOT [58]; our data suggests that observation of actions with the intention to imitate movements can stimulate the recovery of motor control, with significant improvement of abilities in performing common actions, which allows patients to reach maximum independence and quality of life.

The feasibility of the clinical rehabilitative protocol, as demonstrated by the absence of dropouts, allows the treatment of a large cohort of patients with moderate to severe upper limb paresis. The study design was intended to increase the reliability of our findings and the potential applicability to the population of stroke patients as well as to focus on the early phase of stroke recovery; these are important features of this pilot study and make our research potentially useful to clinical practice.

However, our pilot study was restricted by the absence of a follow-up evaluation, which might help to check for the persistence of motor and functional effects. Despite the homogeneity of the sample in terms of type of vascular accident (only pure ischemic lesions) and cognitive functioning, further investigations need a higher sample size to draw any definitive conclusions on the efficacy of this treatment.

## 5. Conclusions

Based on the preliminary study findings, AOT is a promising treatment for upper limb and functional recovery in individuals with moderate to severe upper limb impairment after stroke, due to the feasibility of the treatment and the lack of side effects in this research. Action observation training might become a useful strategy in rehabilitation programs of sub-acute stroke patients, as it improves functionality of the paretic upper limb and allows the recovery of maximum independence in daily life activities. Even if the sample we studied was small, our results seem to suggest that AOT, in addition to conventional rehabilitative treatment in an early phase after stroke, may significantly promote motor recovery and functionality of the paretic upper limb in a sample of stroke patients when compared to TOT. Our results give further relevance to the procedure proposed by AOT, as it proposes even more complex motor sequences with respect to the usual use of the objects. In fact, the AOT movement sequences are standard, regardless of the patient’s level of functionality, whereas in task-oriented training, the level of difficulty is decided by the therapist in relation to the functional level of the patients.

Further clinical trials need to be conducted, enhancing the application of AOT by the use of novel technology, in tele-rehabilitation protocols in the early stages of stroke care.

## Figures and Tables

**Table 1:** Demographic and clinic characteristics of the sample at baseline (n = 32).

Characteristics	All	CG	EG	*p*
	*n* (%)	*n* (%)	*n* (%)	
Subjects	32	16	16	
Dropouts	0 (0)	0 (0)	0 (0)	
Compliants	32 (100)	16 (100)	16 (100)	
Lesion lateralization				
*Right*	17 (53.1)	9 (56.2)	8 (50)	0.04
*Left*	15 (46.9)	7 (43.7)	8 (50)	0.04
**Lesion location (Bamford)**				
*LACI*	18 (56.2)	7 (43.7)	11 (68.7)	0.17
*PACI*	4 (12.5)	2 (12.5)	2 (12.5)	0.17
*POCI*	1 (3.1)	1 (6.2)	0 (0)	0.17
*TACI*	9 (28.1)	6 (37.5)	3 (18.7)	0.17
	**Median (IQR)**	**Median (IQR)**	**Median (IQR)**	
**Age, years**	68.5 (19)	76.5 (13.7)	64.5 (15.75)	0.16
**Gender**	20 males 12 females	10 males 6 females	10 males 6 females	0.6
**Time from onset**	13 (12.75)	20.5 (16.75)	12.5 (6.5)	0.12
** MMSE **	28.5 (1.5)	28 (1.25)		0.97
** CNS **	7.5 (2)	8 (2.75)	7.5 (2)	0.8

**Table 2:** Median, interquartile range (IQR), and significance of pre-treatment and post-treatment scores and of rehabilitative gain in the experimental group and in the control group.

	EG			CG			Rehabilitative Gain		
	T0	T1	*p*	T0	T1	*p*	EG	CG	*p*
BBT									
Paretic Arm	0 (1.75)	10 (22.75)	**0.003**	0 (10)	2.5 (31)	**0.01**	0.1 (0.1)	0 (0.1)	0.30
FMA-UE									
Motor Function	27 (15.25)	47.5 (25.5)	**0.000**	35.5 (23)	39.5 (31)	**0.005**	0.52 (0.52)	0.2 (0.53)	**0.03**
MAS	0 (0)	0 (0)	0.45	1.5 (3)	1.5 (3.75)	0.25	0 (0)	0 (0.1)	0.61
FIM	62 (30.5)	103.5 (32.25)	**0.000**	60 (26.5)	81.5 (43.25)	**0.001**	0.6 (0.38)	0.25 (0.28)	**0.003**

Legend: CG = Control Group; EG = Experimental Group; BBT = Box and Block Test; FMA-UE = Fugl-Meyer Assessment for Upper Extremity; FIM = Functional Independence Measure; MAS = Modified Ashworth Scale. Significant *p*-values are shown in bold.

**Table 3:** Difference scores between baseline and post-treatment assessments in the experimental group and control group for the Fugl-Meyer Assessment for Upper Extremity (FMA-UE) and the Functional Independence Measure (FIM) scales.

	FMA-UE		FIM	
	**EG**	**CG**	**EG**	**CG**
Pt 1	**20**	0	**25**	6
Pt 2	**14**	1	**29**	16
Pt 3	**23**	**14**	**36**	**36**
Pt 4	**21**	**16**	**47**	**41**
Pt 5	**14**	0	**24**	0
Pt 6	5	−6	**51**	10
Pt 7	2	3	20	6
Pt 8	**20**	8	**104**	10
Pt 9	2	6	**23**	14
Pt 10	**18**	−1	**29**	0
Pt 11	**23**	6	**43**	**29**
Pt 12	**15**	3	11	**23**
Pt 13	**10**	**14**	**37**	**50**
Pt 14	6	**19**	**30**	**23**
Pt 15	**12**	7	19	**37**
Pt 16	**22**	8	**55**	18

Patients that overcame the minimum clinically important difference (MCID) for FMA-UE (MCID = 9) and for FIM (MCID = 22) are shown in bold.

## Data Availability

The data that support the findings of this study are available from the corresponding author, [Mancuso Mauro, m.mancuso62@gmail.com], upon reasonable request.

## References

[1] Thrift A.G., Thayabaranathan T., Howard G., Howard V.J., Rothwell P.M., Feigin V.L., Norrving B., Donnan G.A., Cadilhac D. (2017). Global stroke statistics. Int. J. Stroke.

[2] Alawieh A., Zhao J., Feng W. (2018). Factors affecting post-stroke motor recovery: Implications on neurotherapy after brain injury. Behav. Brain Res..

[3] Viosca E., Lafuente R., Martínez J.L., Almagro P.L., Gracia A., González C. (2005). Walking recovery after an acute stroke: Assessment with a new functional classification and the Barthel Index. Arch. Phys. Med. Rehabil..

[4] Bailey R.R., Klaesner J.W., Lang C.E. (2015). Quantifying real-world upper-limb activity in nondisabled adults and adults with chronic stroke. Neurorehabil. Neural Repair.

[5] Franceschini M., Goffredo M., Pournajaf S., Paravati S., Agosti M., De Pisi F., Galafate D., Posteraro F. (2018). Predictors of activities of daily living outcomes after upper limb robot-assisted therapy in subacute stroke patients. PLoS ONE.

[6] Pollock A., Farmer S.E., Brady M.C., Langhorne P., Mead G.E., Mehrholz J., van Wijck F. (2014). Interventions for improving upper limb function after stroke. Cochrane Database Syst. Rev..

[7] Jokinen H., Melkas S., Ylikoski R., Pohjasvaara T., Kaste M., Erkinjuntti T., Hietanen M. (2015). Post-stroke cognitive impairment is common even after successful clinical recovery. Eur. J. Neurol..

[8] Anderlini D., Wallis G., Marinovic W. (2019). Language as a predictor of motor recovery: The case for a more global approach to stroke rehabilitation. Neurorehabil. Neural Repair.

[9] Chen P., Hreha K., Kong Y., Barrett A.M. (2015). Impact of spatial neglect on stroke rehabilitation: Evidence from the setting of an inpatient rehabilitation facility. Arch. Phys. Med. Rehab..

[10] Sun J.H., Tan L., Yu J.T. (2014). Post-stroke cognitive impairment: Epidemiology, mechanisms and management. Ann. Transl. Med..

[11] Garrison K.A., Aziz-Zadeh L., Wong S.W., Liew S.L., Winstein C.J. (2013). Modulating the motor system by action observation after stroke. Stroke.

[12] Zhu M.H., Wang J., Gu X.D., Shi M.F., Zeng M., Wang C.Y., Chen Q.Y., Fu J.M. (2015). Effect of action observation therapy on daily activities and motor recovery in stroke patients. Int. J. Nurs. Sci..

[13] Sale P., Franceschini M. (2012). Action observation and mirror neuron network: A tool for motor stroke rehabilitation. Eur. J. Phys. Rehabil. Med..

[14] Abbruzzese G., Avanzino L., Marchese R., Pelosin E. (2015). Action observation and motor imagery: Innovative cognitive tools in the rehabilitation of Parkinson’s disease. Parkinsons Dis..

[15] Rizzolatti G., Fabbri-Destro M., Cattaneo L. (2009). Mirror neurons and their clinical relevance. Nat. Clin. Pract. Neurol..

[16] Buccino G., Vogt S., Ritzl A., Fink G.R., Zilles K., Freund H.J., Rizzolatti G. (2004). Neural circuits underlying imitation learning of hand actions: An event-related fMRI study. Neuron.

[17] Zhang B., Kan L., Dong A., Zhang J., Bai Z., Xie Y., Liu Q., Peng Y. (2019). The effects of action observation training on improving upper limb motor functions in people with stroke: A systematic review and meta-analysis. PLoS ONE.

[18] Buccino G. (2014). Action observation treatment: A novel tool in neurorehabilitation. Philos. Trans. R. Soc. Lond. B Biol. Sci..

[19] Mattar A.A., Gribble P.L. (2005). Motor learning by observing. Neuron.

[20] Celnik P., Webster B., Glasser D.M., Cohen L.G. (2008). Effects of action observation on physical training after stroke. Stroke.

[21] Zhang X., de Beukelaar T.T., Possel J., Olaerts M., Swinnen S.P., Woolley D.G., Eenderoth N. (2011). Movement observation improves early consolidation of motor memory. J. Neurosci..

[22] Da Silva E.S.M., Ocamoto G.N., Santos-Maia G.L.D., de Fátima Carreira Moreira Padovez R., Trevisan C., de Noronha M.A., Pereira N.D., Borstad A., Russo T.L. (2020). The Effect of Priming on Outcomes of Task-Oriented Training for the Upper Extremity in Chronic Stroke: A Systematic Review and Meta-analysis. Neurorehabil. Neural Repair.

[23] Mang C.S., Campbell K.L., Ross C.J., Boyd L.A. (2013). Promoting neuroplasticity for motor rehabilitation after stroke: Considering the effects of aerobic exercise and genetic variation on brain derived neurotrophic factor. Phys. Ther..

[24] Silva S.M., Corrêa F.I., Faria C.D., Buchalla C.M., Silva P.F., Corrêa J.C. (2015). Evaluation of post-stroke functionality based on the International Classification of Functioning, Disability, and Health: A proposal for use of assessment tools. J. Phys. Ther. Sci..

[25] Steven A. (2005). Julious: Sample size of 12 per group rule of thumb for a pilot study. PHARMACEUTICAL STATISTICS Pharmceut. Statistics.

[26] De Renzi A., Vignolo L.A. (1962). Token test: A sensitive test to detect receptive disturbances in aphasics. Brain.

[27] Spinnler H., Tognoni G. (1987). Taratura e standardizzazione italiana di test neuropsicologici (Italian norms for a battery of neuropsychological tests). Ital. J. Neurol. Sci..

[28] Wilson B., Cockburn J., Halligan P. (1987). Development of a behavioral test of visuospatial neglect. Arch. Phys. Med. Rehabil..

[29] De Renzi E., Pieczuro A., Vignolo L.A. (1968). Ideational apraxia: A quantitative study. Neuropsychologia.

[30] Measso G., Cavarzeran F., Zappala G., Lebowitz B.D., Crook T.H., Pirozzolo F.J., Pirozzolo L.A., Massari D., Grigoletto F. (1993). The mini-mental state examination: Normative study of an Italian random sample. Dev. Neuropsychol..

[31] Brott T., Adams H.P., Olinger C.P., Marler J.R., Barsan W.G., Biller J., Spilker J., Holleran R., Eberle E., Hertzberg V. (1989). Measurements of acute cerebral infarction: A clinical examination scale. Stroke.

[32] Côté R., Hachinski V.C., Shurvell B.L., Norris J.W., Wolfson C. (1986). The Canadian Neurological Scale: A preliminary study in acute stroke. Stroke.

[33] Côté R., Battista R.N., Wolfson C., Boucher J., Adam J., Hachinski V. (1989). The Canadian Neurological Scale: Validation and reliability assessment. Neurology.

[34] Bamford J., Sandercock P., Dennis M., Burn J., Warlow C. (1991). Classification and natural history of clinically identifiable subtypes of cerebral infarction. Lancet.

[35] Fugl-Meyer A.R., Jääskö L., Leyman I., Olsson S., Steglind S. (1975). The post-stroke hemiplegic patient. 1. A method for evaluation of physical performance. Scand. J. Rehabil. Med..

[36] Hsieh Y.W., Wu C.Y., Lin K.C., Chang Y.F., Chen C.L., Liu J.S. (2009). Responsiveness and validity of three outcome measures of motor function after stroke rehabilitation. Stroke.

[37] Fu T.S.T., Wu C.Y., Lin K.C., Hsieh C.J., Liu J.S., Wang T.N., Ou-Yang P. (2012). Psychometric comparison of the shortened Fugl-Meyer Assessment and the streamlined Wolf Motor Function Test in stroke rehabilitation. Clin. Rehabil..

[38] Narayan Arya K., Verma R., Garg R.K. (2011). Estimating the minimal clinically important difference of an upper extremity recovery measure in subacute stroke patients. Top. Stroke Rehabil..

[39] Desrosiers J., Bravo G., Hébert R., Dutil É., Mercier L. (1994). Validation of the Box and Block Test as a measure of dexterity of elderly people: Reliability, validity, and norms studies. Arch. Phys. Med. Rehabil..

[40] Mathiowetz V., Volland G., Kashman N., Weber K. (1985). Adult norms for the Box and Block Test of manual dexterity. Am. J. Occup. Ther..

[41] Lin K.C., Chuang L.L., Wu C.Y., Hsieh Y.W., Chang W.Y. (2010). Responsiveness and validity of three dexterous function measures in stroke rehabilitation. J. Rehabil. Res. Dev..

[42] Chen H.M., Chen C.C., Hsueh I.P., Huang S.L., Hsieh C.L. (2009). Test-retest reproducibility and smallest real difference of 5 hand function tests in patients with stroke. Neurorehabil. Neural Repair.

[43] Chumney D., Nollinger K., Shesko K., Skop K., Spencer M., Newton R.A. (2010). Ability of Functional Independence Measure to accurately predict functional outcome of stroke-specific population: Systematic review. J. Rehabil. Res. Dev..

[44] Glenny C., Stolee P. (2009). Comparing the functional independence measure and the interRAI/MDS for use in the functional assessment of older adults: A review of the literature. BMC Geriatr..

[45] Wallace D., Duncan P.W., Lai S.M. (2002). Comparison of the responsiveness of the Barthel Index and the motor component of the Functional Independence Measure in stroke: The impact of using different methods for measuring responsiveness. J. Clin. Epidemiol..

[46] Beninato M., Gill-Body K.M., Salles S., Stark P.C., Black-Schaffer R.M., Stein J. (2006). Determination of the minimal clinically important difference in the FIM instrument in patients with stroke. Arch. Phys. Med. Rehabil..

[47] Bohannon R.W., Smith M.B. (1987). Interrater reliability of a modified Ashworth scale of muscle spasticity. Phys. Ther..

[48] Van Wijck F.M., Pandyan A.D., Johnson G.R., Barnes M.P. (2001). Assessing motor deficits in neurological rehabilitation: Patterns of instrument usage. Neurorehabil. Neural Repair.

[49] Shaw L., Rodgers H., Price C., van Wijck F., Shackley P., Steen N., Barnes M., Ford G., Graham L. (2010). BoTULS: A multicentre randomised controlled trial to evaluate the clinical effectiveness and cost-effectiveness of treating upper limb spasticity due to stroke with botulinum toxin type A. Health Technol. Assess..

[50] Franceschini M., Ceravolo M.G., Agosti M., Cavallini P., Bonassi S., Dall’Armi V., Massucci M., Schifini F., Sale P. (2012). Clinical relevance of action observation in upper-limb stroke rehabilitation: A possible role in recovery of functional dexterity. A randomized clinical trial. Neurorehabil. Neural Repair.

[51] Make B. (2007). How can we assess outcomes of clinical trials: The MCID approach. COPD.

[52] Rizzolatti G., Sinigaglia C. (2016). The mirror mechanism: A basic principle of brain function. Nat. Rev. Neurosci..

[53] Brunner I.C., Skouen J.S., Ersland L., Grüner R. (2014). Plasticity and response to action observation: A longitudinal FMRI study of potential mirror neurons in patients with subacute stroke. Neurorehabil. Neural Repair.

[54] Gatti R., Tettamanti A., Gough P.M., Riboldi E., Marinoni L., Buccino G. (2013). Action observation versus motor imagery in learning a complex motor task: A short review of literature and a kinematics study. Neurosci. Lett..

[55] Kim C.H., Bang D.H. (2016). Action observation training enhances upper extremity function in subacute stroke survivor with moderate impairment: A double-blind, randomized controlled pilot trial. J. Korean Soc. Phys. Med..

[56] Gladstone D.J., Danells C.J., Black S.E. (2002). The Fugl-Meyer assessment of motor recovery after stroke: A critical review of its measurement properties. Neurorehabil. Neural Repair.

[57] Platz T., Pinkowski C., van Wijck F., Kim I.H., Di Bella P., Johnson G. (2005). Reliability and validity of arm function assessment with standardized guidelines for the Fugl-Meyer Test, Action Research Arm Test and Box and Block Test: A multicentre study. Clin. Rehabil..

[58] Hsieh Y.W., Lin Y.H., Zhu J.D., Wu C.Y., Lin Y.P., Chen C.C. (2020). Treatment Effects of Upper Limb Action Observation Therapy and Mirror Therapy on Rehabilitation Outcomes after Subacute Stroke: A Pilot Study. Behav. Neurol..

[59] Fu J., Zeng M., Shen F., Cui Y., Zhu M., Gu X., Sun Y. (2017). Effects of action observation therapy on upper extremity function, daily activities and motion evoked potential in cerebral infarction patients. Medicine.

[60] Zhu J.D., Cheng C.H., Tseng Y.J., Chou C.C., Chen C.C., Hsieh Y.W., Liao Y.H. (2019). Modulation of Motor Cortical Activities by Action Observation and Execution in Patients with Stroke: An MEG Study. Neural Plast..

[61] Peng T.H., Zhu J.D., Chen C.C., Tai R.Y., Lee C.Y., Hsieh Y.W. (2019). Action observation therapy for improving arm function, walking ability, and daily activity performance after stroke: A systematic review and meta-analysis. Clin. Rehabil..

[62] Oh S.J., Lee J.H., Kim D.H. (2019). The effects of functional action-observation training on gait function in patients with post-stroke hemiparesis: A randomized controlled trial. Technol. Health Care.

